# Associations between food-specific IgG antibodies and intestinal permeability biomarkers

**DOI:** 10.3389/fnut.2022.962093

**Published:** 2022-09-06

**Authors:** Alexandra Adorno Vita, Heather Zwickey, Ryan Bradley

**Affiliations:** ^1^Helfgott Research Institute, National University of Natural Medicine, Portland, OR, United States; ^2^Herbert Wertheim School of Public Health and Human Longevity Sciences, University of California, San Diego, La Jolla, CA, United States

**Keywords:** permeability, food sensitivity, occludin, vinculin, lipopolisaccharide, cytolethal distending toxin (CdtB)

## Abstract

Increasing translational evidence suggests that intestinal permeability may be a contributing factor to systemic inflammatory events and numerous pathologies. While associations between IgE-mediated food allergies and increased intestinal permeability have been well-characterized, the relationship between IgG-mediated food sensitivities and intestinal permeability is not well-described in the literature. Thus, we tested for associations between intestinal permeability biomarkers and food-specific IgG antibodies in 111 adults, with and without gastrointestinal symptoms. All biomarkers and food-specific IgG antibodies were measured *via* ELISA. The intestinal permeability biomarkers anti-lipopolysaccharide (LPS) and anti-occludin IgG and IgA antibodies, but not anti-vinculin or anti-CdtB IgG antibodies, were significantly and positively associated with IgG-mediated food sensitivities. These significant relationships were attenuated by adjusting for the severity of wheat, dairy, and egg reactions. The results of this study support strong associations between titers of food-specific IgG antibodies and intestinal permeability biomarkers in adults, to the extent that the presence of multiple IgG antibodies to food, and increasing IgG food titers, can be considered indicative of increased antibodies to LPS and occludin. Notably, neither IgG titers to wheat, eggs, and dairy, nor permeability biomarkers, were increased in symptomatic participants compared to those without symptoms.

## Introduction

The development of immunologic tolerance to orally-introduced molecules is critical to prevention of food-related allergies, and is achieved through interactions between food-specific antigens and cells of the mucosal immune system under specific conditions (1). A key mechanism of immunologic tolerance is through the induction of regulatory T cells (T_*regs*_), which suppress the activity of CD4^+^ T helper (Th) cells and other immune cells through direct (e.g., cell-cell interactions) and indirect mechanisms (e.g., suppressive cytokines IL-10 and transforming growth factor (TGF) beta) (1–3). As such, T_*regs*_ create an inhibitory environment where immune cells may interact with food-specific antigens without becoming activated, thus tolerizing those cells to said antigens.

While food *allergies* are classic mast cell and IgE-mediated immune reaction that cause immediate hypersensitivity responses (4, 5), food *sensitivities* are IgG-mediated immune reactions that cause delayed-type hypersensitivity responses (6). IgG-mediated sensitivities against food antigens can arise due to repeated exposure and a lack of inhibition, which may increase the probability of food-specific antigens coming in contact with their antigen-specific T and/or B cell in an inflammatory environment. This subsequently results in the unchecked activation and proliferation of those cells through loss of tolerance mechanisms similar to those which are implicated in the generation of autoantibodies against self-tissue (2, 3, 6).

The intestinal barrier is an important consideration in immune-mediated reactions to food antigens, as it serves the important function of being a selectively permeable barrier between environmental exposures and systemic circulation. Permeability of the paracellular space between enterocytes is strictly controlled by various tight junction (e.g., occludin and claudin), adherens junction (e.g., cadherin), and desmosomal proteins, which work together to ensure that only specific molecules can pass into systemic circulation (7, 8). When the integrity of this barrier is compromised, increased intestinal permeability can be identified through serum biomarkers, such as anti-occludin IgA, IgG, and IgM; anti-lipopolysaccharide (LPS) IgA, IgG, and IgM; anti-cytolethal distending toxin B (CdtB) IgG; and anti-vinculin IgG, among others (9–12).

Antibodies generated against structural components of the intestinal barrier, such as occludin and vinculin (7, 13), may flag these proteins for destruction and further compromise the integrity of enterocyte tight junctions. Subsequently, bacterial components or products that would otherwise be restricted to the gut, such as LPS and CdtB, can translocate across the intestinal barrier and enter systemic circulation (14, 15). Although there is no formal diagnosis of intestinal permeability, the aforementioned molecules act as biomarkers for increased permeability due to their physiological relevance to barrier function.

While associations between IgE-mediated food allergies and increased intestinal permeability have been suggested in the literature (16), potential associations between IgG-mediated food sensitivities and increased permeability is less well-described. Thus, this study tested for associations between intestinal permeability biomarkers and IgG antibodies specific to a variety of foods, with and without adjustment for potential confounders.

## Materials and methods

### Participants

This study was reviewed and approved by the Institutional Review Board of the National University of Natural Medicine (IRB # 102114) and conducted in accordance with the Declaration of Helsinki. All participants provided their written informed consent to participate in the study. Participants were recruited for the study through community-based recruitment and referral from local clinics. All participants were included if they were age 18–65, willing to provide informed consent, and willing and able to have blood drawn.

Participants were excluded if they had a diagnosis of stroke, brain tumor, hydrocephalus, epilepsy, other degenerative disorders, or traumatic brain injury; History of cancer (other than basal and squamous cell skin cancers), liver disease or hematological disorders in the past 5°years; History of any of autoimmune conditions within the past 5°years, including but not limited to: Diabetes type 1, ulcerative colitis, Crohn’s disease, Celiac disease, Hashimoto’s or autoimmune hypothyroid, Grave’s disease or autoimmune hyperthyroid, multiple sclerosis, polymyalgia rheumatica, psoriasis, rheumatoid arthritis, Sjogren’s disease, systemic lupus erythematosus, reactive arthritis (Reiter’s syndrome), myasthenia gravis, primary biliary cirrhosis, scleroderma, pernicious anemia, vitiligo; Previous diagnosis and treatment for SIBO within the last 6°months; History of fecal matter transplant or helminth therapy within the past 6°months; Current symptoms or history of hypoglycemia or poorly controlled diabetes; Use of antibiotics, immunosuppressants, or hormone therapy in the past 6°months; Use of supplemental berberine, goldenseal, oregano, garlic, or neem in the past 2°months; Currently pregnant or breast feeding.

Although probiotic use and past of IgE-mediated food allergy could potentially impact intestinal permeability, these were not part of our exclusion criteria. However, there were no reports of IgE food allergy in the medical history intakes. Probiotic users were not excluded for feasibility, as we would have also had to exclude candidates who ate yoghurt, fermented foods, apple cider vinegar, some beverages, and, based on current literature regarding *pre*biotics, high fiber consumers as well.

The final dataset contained 111 adults aged 18–64, some of which had a broad range of functional gastrointestinal symptoms but without diagnosed gastrointestinal pathology and others who were asymptomatic for gastrointestinal symptoms. Gastrointestinal symptoms were self-reported and defined as diarrhea, constipation, bloating, or abdominal pain more than once per week (GI symptom survey outlined in section “Health history questionnaires” Survey Data).

### Health history questionnaires

An online health history questionnaire was administered using REDCap software (17, 18). This questionnaire collected data regarding gastrointestinal symptoms, frequency of alcohol consumption, medical history, potential exposure to gastrointestinal microbes, and adverse reactions to foods.

For the assessment of gastrointestinal symptoms, three questions were asked related to the presence, frequency, and severity. First, participants were asked if experienced any of the following symptoms in the past 7°days and were instructed to answer “yes” or “no” to each one: *Diarrhea* (loose, watery or frequent stool > 3/day), *Constipation* (infrequent < 1/day or hard to pass stool), *Both diarrhea and constipation*, and *Well-formed, easy to pass stools* (1–3 times per day).

Second, participants were asked to check the box for the number days per week that they experienced each of the following symptoms on a scale of 0 to 7 (0 = no days per week and 7 = daily): *Bloating* (abdominal fullness or pressure), *Abdominal pain or discomfort, Gas, Burping*, and *Reflux*.

Third, participants were asked to consider the past week and to rate the severity for each of the following symptoms on a scale of 0 to 10 severity (10 = worst, 5 = moderate, 0 = you do not experience the symptom): *Bloating* (abdominal fullness or pressure), *Abdominal pain or discomfort, Gas, Burping*, and *Reflux*.

For the assessment of the frequency of alcohol consumption, participants were asked to indicate the amount of alcohol consumed per week out of the following options: 0 drinks per week, 1–7 drinks per week, 8–14 drinks per week, 15–21 drinks per week, and 22 or more drinks per week.

For the assessment of medical history and to confirm eligibility for the study, participants were asked questions regarding the use of specific medications, medical procedures, and diagnoses (current, past, or never) of medical conditions related to the exclusionary criteria. Participants were also questioned on the presence of IgE food allergies. Finally, to gain information on potential exposure to pathogenic gastrointestinal microbes, participants were asked to answer “yes” or “no” as to whether they have experienced the each of following: *History of stomach flu or food poisoning* and *History of travel to developing countries*.

### Food sensitivity testing

Plasma samples were collected and stored at −80°C until analysis. All food sensitivity testing was performed by Cyrex Laboratories (Phoenix, AZ, United States), and carried out as previously described by Vojdani et al. (19). Briefly, food antigens were prepared in stock solutions at concentrations of 1 mg/mL, diluted 1:50–1:200, and added to ELISA plates at 100 μL per well. The previously described ELISA methods were used to determine participant serum antibody reactivity levels against specific food antigens in comparison with control wells and performed in quadruplicate. An ELISA microplate reader recorded the optic densities (OD) at 405 nm.

### ELISA for detection of anti-LPS and anti-occludin antibodies

Plasma samples were collected and stored at −80°C until analysis. Detection for anti-LPS and anti-occludin IgG, IgA, and IgM antibodies were performed by Cyrex Laboratories (Phoenix, AZ, United States), and were measured *via* ELISA as previously described by Vojdani et al. (11, 12). Briefly, diluted LPS or occludin antigens were added to ELISA microplates at 100 μL per well. Plates containing LPS were incubated for 24 h at 4°C, and plates containing occludin were incubated for 8 h at room temperature followed by an additional 16 h at 4°C. Following incubation, plates underwent rounds of rising; plates containing LPS were rinsed with 5% Tween in PBS, and plates containing occluding were rinsed with 5% Tween 20 in Tris buffered saline (TBS). All plates then underwent blocking with 2% bovine serum albumin to prevent non-specific binding, followed by the addition of diluted human serum samples at 100 μL per well, and further rinsing with TBS. Alkaline phosphatase-labeled anti-human IgG, IgM, or IgA antibodies were added to the wells for detection of specific antibody isotypes. An ELISA microplate reader recorded the optic densities (OD) at 405 nm.

### ELISA for detection of anti-CdtB and anti-vinculin

Plasma samples were collected and stored at −80°C until analysis. The anti-CdtB and anti-vinculin antibody levels were measured by ELISA, using the first generation methodology previously reported by Pimentel et al. (15). Antigens used in the ELISA include complete recombinant *Campylobacter* Cdt protein (Creative Biomart, Shirley, NY, United States) and full length human vinculin protein (Novoprotein, Short Hills, NJ, United States), both at 1.2°μg/mL. The optical densities (OD) were measured at 370°nm.

### Outcome and exposure variables

The outcome variables for all regression models included serum antibody titers of eight intestinal permeability biomarkers: anti-occludin IgA, IgG, and IgM; anti-LPS IgA, IgG, and IgM; anti-CdtB IgG; and anti-vinculin IgG. The intestinal permeability biomarkers were all reported as continuous variables.

The exposure variables in the models were derived from 90 IgG food sensitivity tests for a variety of foods, which were reported both continuously and categorically. The values were reported in three reaction severity categories based on antibody titers, with higher titers corresponding to higher severity. Severity category 1 included negative reactions (values 0–1); severity category 2 included low-positive reactions (values 1–1.2); severity category 3 included positive reactions (values greater than 1.2).

The continuous and categorical results of the food sensitivity tests were then recoded into both summed and weighted variables, which were used as the exposure variables. The summed categorical variable was created by calculating the total number of “severity category 3” tests, or the total number of positive tests, for each participant; as such, this variable was labeled *categorical total positive tests*. To create the summed continuous variable, the total values of “severity category 3” tests, or total values of positive food sensitivity tests, were summed for each participant; as such, this variable was labeled *continuous total positive tests*. Participants without any “severity category 3” tests were not excluded and their summed variable equaled zero.

Given permeability may be associated with both the total number of sensitivities (indicated by our summed variables) as well as severity of the reactions to each food-specific antigen, a severity-weighted variable was created. To do so, food sensitivity results were collapsed into a weighted score calculated by multiplying severity category of the reaction by either the number of foods in each reaction category (*categorical weighted food sensitivity score* variable) or by the values associated with foods in each reaction category (*continuous weighted food sensitivity score* variable). For example, [40 foods in severity category 3] + [20 foods in severity category 2] + [30 in severity category 1] = 190 as a categorical weighted food sensitivity score [*i.e.*, (40*3) + (20*2) + (30*1) = 190]. A similar formula was followed for calculating the continuous weighted score, substituting the total foods within each reaction category for the values associated with foods within each reaction category.

### Covariates

Covariates included in the analyses include *age*, *sex at birth*, *reactive food intake*, and *weighted reactive food sensitivity score*. For the demographic covariates, *age* was reported continuously in years, and *sex* was reported as male or female.

Intake of commonly reactive foods (e.g., eggs, dairy, and wheat) by participants was collected by a survey assessing for the consumption of dairy, eggs, and wheat. For the three food groups, the reporting categories were never, seldom, frequently, and daily. For the new *reactive food intake* variable, never and seldom were combined into a low intake category, and frequently and daily were combined into a high intake category.

The *weighted reactive food sensitivity score* was created in the same manner as the previous weighted food sensitivity score; however, only the hard cheese IgG, soft cheese IgG, yogurt IgG, egg white IgG, egg yolk IgG, and wheat IgG food sensitivity tests were included. These were the specific foods included in the panel that represent the consumption of dairy, eggs, and wheat from the participant survey.

### Statistical analysis

Study data were collected and managed using REDCap electronic data capture tools hosted at National University of Natural Medicine, whereby data was then exported for statistical analysis (17, 18). For comparison of average serum biomarkers between symptomatic and asymptomatic individuals, Mann Whitney *U* tests were performed; variables were not normally distributed.

For the regression analyses, variables were log transformed. Linear regression models were used to test the cross-sectional relationships between IgG food sensitivity test results and the intestinal permeability biomarkers. Multivariate, nested models were created to analyze *total positive tests* exposure variables (*categorical* or *continuous*) and the *weighted food sensitivity score* exposure variables (*categorical* or *continuous*) corresponding to Model n and Model n,’ respectively. For the unadjusted, crude model only the exposure variables were included (Models 1 and 1′) were included. Models 2 and 2′ added adjustment for age and sex. Models 3 and 3′ added adjustment for the intake of commonly reactive foods including wheat, dairy, and eggs. Model 4 and 4′ added adjustment for the weighted reactive food sensitivity scores for wheat, dairy, and eggs. While using continuous variables has a number of advantages in regression analyses, categorical forms of the variables were also utilized due to the common clinical assessment of IgG-mediated food sensitivity test results along categorical delineations. As such, we used the categorical and continuous variables in its own separate regression analysis to confirm that the same relationship was present in both, for a total of four separate regressions. All analyses were run in R version 3.3.2.

## Results

### Descriptive statistics

Baseline characteristics are reported in Table 1.

**TABLE 1:** Baseline characteristics (*n* = 111).

Variable	All participants	Prevalence of GI symptoms	
	Total (n = 111)	Symptomatic (n = 19)	Asymptomatic (n = 92)
**Age**	***M (SD)***	***M (SD)***	***M (SD)***
	37.3 (±11.1)	37.1 (±10.4)	37.3 (±11.2)
**Sex assigned at birth**	***n (%)***	***n (%)***	***n (%)***
Male	27 (24.3%)	2 (10.5%)	24 (26.1%)
Female	84 (75.7%)	17 (89.5%)	68 (73.9%)
**Frequency of alcohol use**	***n (%)***	***n (%)***	***n (%)***
0 times per week	33 (29.7%)	4 (21.0%)	29 (31.5%)
1-7 times per week	69 (62.2%)	9 (47.4%)	60 (65.2%)
8-14 drinks per week	3 (2.7%)	1 (5.3%)	2 (2.2%)
15-21 drinks per week	0 (0%)	0 (0%)	0 (0%)
22 or more drinks per week	1 (.09%)	0 (0%)	1 (1.1%)
No response	5 (4.5%)	5 (26.3%)	0 (0%)
**History of GI pathogen exposure**	***n (%)***	***n (%)***	***n (%)***
GI Infection	44 (39.6%)	10 (52.6%)	34 (37.0%)
Travel to developing country	55 (49.5%)	9 (47.4%)	46 (50%)

Shown are the baseline characteristics of study participants.

### Comparison of antibody concentrations between symptomatic and asymptomatic individuals

There were no significant differences in the average values of permeability biomarkers between symptomatic and asymptomatic individuals (Table 2). Additionally, there were no significant differences in the IgG antibody titers of common reactive foods (e.g., wheat, eggs, and dairy) between symptomatic and asymptomatic individuals (Table 2).

**TABLE 2:** Difference in average biomarker concentrations between symptomatic and asymptomatic participants (*n* = 111).

Variable	Asymptomatic	Symptomatic	Mann-whitney *U* test			
	Mean (SD)	Mean (SD)	U	Wilcoxon W rank sum	SE	*P*-value
**Anti-LPS**						
IgG	0.8858 (0.02856)	0.9321 (0.07054)	938.500	1128.500	127.710	0.614
IgM	0.6563 (0.03627)	7537 (0.08912)	1001.000	1191.000	127.706	0.320
IgA	1.0672 (0.04906)	1.0189 (0.11229)	792.000	982.000	127.712	0.521
**Anti-occludin**						
IgG	0.8460 (0.03143)	0.8332 (0.08085)	815.000	1005.000	127.703	0.644
IgM	0.7038 (0.03675)	0.663 (0.05826)	849.500	1039.500	127.701	0.848
IgA	0.8414 (0.03288)	0.8274 (0.06025)	853.000	1043.000	127.712	0.869
**Anti-vinculin**						
IgG	1.0337 (0.80878)	1.0167 (0.76145)	854.000	1044.000	122.653	0.883
**Anti-CdtB**						
IgG	2.4531 (0.88295)	2.4477 (0.81774)	795.500	985.500	122.669	0.741
**Anti-wheat**						
IgG	0.8758 (0.04077)	0.9821 (0.10102)	982.500	1172.500	127.687	0.395
IgA	0.7935 (0.03700)	0.9311 (0.09419)	1015.000	1205.000	127.713	0.270
**Anti-egg**						
IgG (yolk)	0.8572 (0.05404)	0.8968 (0.12130)	922.500	1112.500	127.716	0.704
IgG (white)	1.2580 (0.08086)	1.2537 (0.17870)	989.000	1088.000	127.720	0.851
IgA (yolk)	0.6738 (0.03186)	0.7616 (0.08497)	990.500	1180.500	127.704	0.362
IgA (white)	0.5782 (0.03762)	0.6342 (0.10020)	844.500	1034.500	127.673	0.817
**Anti-cheese**						
IgG	0.9062 (0.05305)	1.0021 (0.13763)	931.500	1121.500	127.717	0.653
IgA	0.9323 (0.07342)	0.8789 (0.15737)	821.500	1011.500	127.713	0.681

Shown are Mann-Whitney *U* Test results comparing average antibody concentrations of permeability biomarkers and highly reactive foods between symptomatic and asymptomatic individuals. No significant differences were observed.

### Associations between total positive tests and intestinal permeability biomarkers

A significant association was evident between the categorical total positive tests variable and anti-LPS IgG and anti-occludin IgG antibody titers (Table 3). The relationship was weakened and no longer significant in Model 4 when adjusted for the severity of the reaction to wheat, eggs, and dairy. Similarly, continuous total positive tests variable was significantly associated with titers of anti-LPS IgG and IgA and anti-occludin IgG and IgA (Table 4). However, unlike what was observed in the categorical models (Table 3), the significant association between continuous total positive tests and anti-LPS IgG and IgA, and anti-occludin IgA, was not weakened in by the adjustment for adjusted for the severity of the reaction to wheat, eggs, and dairy in Model 4; significance was only attenuated in Model 4 for anti-occludin IgG (Table 4).

**TABLE 3:** Associations between intestinal permeability biomarkers and categorical summed food sensitivity measures (*n* = 111).

Exposure: categorical total positive tests	LPS IgA β [95% CI] *p*-value	LPS IgG β [95% CI] *p*-value	LPS IgM β [95% CI] *p*-value	Occludin IgA β [95% CI] *p*-value	Occludin IgG β [95% CI] *p*-value	Occludin IgM β [95% CI] *p*-value	CdtB IgG β [95% CI] *p*-value	Vinculin IgG β [95% CI] *p*-value
Model 1′	0.0046 [0.001, 0.01] (*p* = 0.11)	0.0049 [0.0015, 0.0083] (*p* = 0.005)*	0.0022 [−0.0043, 0.0087] (*p* = 0.5)	0.004 [−0.0003, 0.0083] (*p* = 0.068)	0.0048 [0.00028, 0.0094] (*p* = 0.038)*	0.0039 [−0.0021, 0.01] (*p* = 0.19)	−0.0046 [−0.016, 0.006] (*p* = 0.41)	−0.0066 [0.017, 0.0036] (*p* = 0.20)
Model 2′	0.0039 [−0.0016, 0.0094] (*p* = 0.16)	0.0048 [0.0014, 0.0083] (*p* = 0.01)*	0.0024 [−0.0036, 0.0084] (*p* = 0.43)	0.0037 [−0.0006, 0.008] (*p* = 0.09)	0.0052 [0.0006, 0.0098] (*p* = 0.03)*	0.0038 [−0.002, 0.0096] (*p* = 0.2)	−0.0043 [−0.016, 0.007] (*p* = 0.45)	−0.0063 [−0.017, 0.004] (*p* = 0.23)
Model 3′	0.003 [−0.0027, 0.0087] (*p* = 0.30)	0.0046 [0.0011, 0.0082] (*p* = 0.01)*	0.0031 [−0.0031, 0.0094] (*p* = 0.32)	0.003 [−0.0014, 0.0074] (*p* = 0.18)	0.0041 [−0.0006, 0.009] (*p* = 0.09)	0.0045 [−0.0016, 0.011] (*p* = 0.15)	−0.0052 [-0.017, 0.0068] (*p* = 0.39)	−0.007 [−0.017, 0.0043] (*p* = 0.23)
Model 4′	0.0043 [−0.003, 0.012] (*p* = 0.24)	0.0016 [−0.0029, 0.006] (*p* = 0.48)	0.0026 [−0.0054, 0.011] (*p* = 0.52)	0.0033 [0.0023, 0.009] (*p* = 0.24)	−0.0016 [0.0074, 0.0041] (*p* = 0.56)	0.0047 [−0.0028, 0.013] (*p* = 0.213)	−0.0084 [−0.046, 0.0069] (*p* = 0.28)	−0.003 [0.087, 0.039] (*p* = 0.64)

Model 1 = Intestinal permeability biomarker only; Model 2 = Model 1 + age + sex; Model 3 = Model 2 + consumption of wheat, dairy, and eggs; Model 4 = Model 3 + severity of the reaction to wheat, dairy, and eggs. (p-value < 0.05)*.

**TABLE 4:** Associations between intestinal permeability biomarkers and continuous summed food sensitivity measures (*n* = 111).

Exposure: continuous total positive tests	LPS IgA β [95% CI] *p*-value	LPS IgG β [95% CI] *p*-value	LPS IgM β [95% CI] *p*-value	Occludin IgA β [95% CI] *p*-value	Occludin IgG β [95% CI] *p*-value	Occludin IgM β [95% CI] *p*-value	CdtB IgG β [95% CI] *p*-value	Vinculin IgG β [95% CI] *p*-value
Model 1′	0.0044 [0.001, 0.0078] (*p* = 0.01)*	0.0051 [0.0031, 0.0071] (*p* < 0.001)*	0.0019 [−0.0021, 0.0059] (*p* = 0.35)	0.0036 [0.001, 0.0062] (*p* = 0.007)*	0.0057 [0.003, 0.0084] (*p* < 0.001)*	0.0021 [−0.0017, 0.0058] (*p* = 0.28)	−0.0007 [0.0077, 0.0062] (*p* = 0.84)	−0.0039 [−0.01, 0.0024] (*p* = 0.22)
Model 2′	0.004 [0.00063, 0.0073] (*p* = 0.03)*	0.0051 [0.0031, 0.0071] (*p* < 0.001)*	0.0016 [−0.0021, 0.0054] (*p* = 0.38)	0.0033 [0.0073, 0.006] (*p* = 0.013)*	0.0058 [0.0032, 0.0085] (*p* < 0.001)*	0.0018 [−0.0019, 0.0054] (*p* = 0.33)	−0.0005 [0.0076, 0.0066] (*p* = 0.88)	−0.0039 [−0.01, 0.0025] (*p* = 0.23)
Model 3′	0.0036 [0.00007, 0.0074] (*p* = 0.046)*	0.005 [0.003, 0.0071] (*p* < 0.001)*	0.0022 [−0.017, 0.0061] (*p* = 0.26)	0.003 [0.00022, 0.0057] (*p* = 0.03)*	0.0054 [0.0026, 0.0082] (*p* < 0.001)*	0.0023 [−0.0015, 0.00061] (*p* = 0.24)	−0.0008 [−0.0082, 0.0066] (*p* = 0.83)	−0.0039 [−0.011, 0.0029] (*p* = 0.26)
Model 4′	0.0064 [0.0016, 0.011] (*p* = 0.009)*	0.0047 [0.0018, 0.0076] (*p* = 0.001)*	0.0022 [−0.0032, 0.0076] (*p* = 0.43)	0.0044 [0.0006, 0.0081] (*p* = 0.02)*	0.0034 [−0.00045, 0.0072] (*p* = 0.83)	0.0024 [−0.0028, 0.0077] (*p* = 0.36)	−0.0008 [−0.012, 0.0087] (*p* = 0.77)	−0.641 [−4.83, 3.55] (*p* = 0.76)

Model 1 = Intestinal permeability biomarker only; Model 2 = Model 1 + age + sex; Model 3 = Model 2 + consumption of wheat, dairy, and eggs; Model 4 = Model 3 + severity of the reaction to wheat, dairy, and eggs. (p-value < 0.05)*.

### Associations between weighted food sensitivity score and intestinal permeability biomarkers

There was a significant association between categorical weighted food sensitivity and anti-LPS IgG and anti-occludin IgG antibody titers in (Table 5). This significance was attenuated in Model 4′ when adjusted for the severity of the reaction to wheat, eggs, and dairy (Table 5). In the continuous weighted food sensitivity model (Table 6), there were significant associations with titers of anti-LPS IgG and IgA, and with anti-occludin IgG and IgA. The associations observed were weakened in Model 4′ and no longer significant when adjusted for the severity of the reaction to wheat, eggs, and dairy (Table 6).

**TABLE 5:** Association between intestinal permeability biomarkers and categorical weighted food sensitivity score (*n* = 111).

Exposure: categorical weighted food sensitivity score	LPS IgA β [95% CI] *p*-value	LPS IgG β [95% CI] *p*-value	LPS IgM β [95% CI] *p*-value	Occludin IgA β [95% CI] *p*-value	Occludin IgG β [95% CI] *p*-value	Occludin IgM β [95% CI] *p*-value	CdtB IgG β [95% CI] *p*-value	Vinculin IgG β [95% CI] *p*-value
Model 1′	0.002 [−0.0004, 0.0043] (*p* = 0.10)	0.0021 [0.00065,0.0035] (*p* = 0.005)*	0.0009 [−0.0018, 0.0036] (*p* = 0.52)	0.0015 [−0.00034, 0.0033] (*p* = 0.11)	0.0022 [0.00027, 0.0041] (*p* = 0.03)*	0.0017 [−0.0008, 0.0043] (*p* = 0.18)	−0.0027 [−0.0074, 0.002] (*p* = 0.25)	−0.0031 [−0.0074, 0.0011] (*p* = 0.148)
Model 2′	0.0016 [−0.00075, 0.0039] (*p* = 0.18)	0.0021 [0.00061, 0.0035] (*p* = 0.006)*	0.00086 [−0.0017, 0.0034] (*p* = 0.502)	0.0014 [−0.00046, 0.0032] (*p* = 0.142)	0.0024 [0.00044, 0.0043] (*p* = 0.02)*	0.0016 [−0.00087, 0.0041] (*p* = 0.2)	−0.0026 [−0.0073, 0.0022] (*p* = 0.288)	−0.003 [−0.007, 0.0013] (*p* = 0.175)
Model 3′	0.0012 [−0.0012, 0.0036] (*p* = 0.32)	0.002 [0.00051, 0.0035] (*p* = 0 .009)*	0.0012 [−0.0014, 0.0036] (*p* = 0.36)	0.0011 [−0.00078, 0.003] (*p* = 0.25)	0.002 [0.00002, 0.004] (*p* = 0.047)*	0.0019 [−0.00063, 0.0045] (*p* = 0.14)	−0.0031 [−0.0081, 0.0016] (*p* = 0.22)	−0.0032 [−0.0078, 0.0013] (*p* = 0.17)
Model 4′	0.0017 [−0.0013, 0.0048] (*p* = 0.26)	0.00078 [−0.0011, 0.0026] (*p* = 0.405)	0.00093 [−0.0024, 0.0042] (*p* = 0.58)	0.0011 [−0.0012, 0.0035] (*p* = 0.35)	−0.00019 [−0.0026, 0.0022] (*p* = 0.877)	0.0021 [−0.0011, 0.0053] (*p* = 0.2)	−0.0049 [−0.035, 0.0013] (*p* = 0.12)	−0.0021 [−0.081, 0.043] (*p* = 0.46)

Model 1 = Intestinal permeability biomarker only; Model 2 = Model 1 + age + sex; Model 3 = Model 2 + consumption of wheat, dairy, and eggs; Model 4 = Model 3 + severity of the reaction to wheat, dairy, and eggs. (p-value < 0.05)*.

**TABLE 6:** Association between intestinal permeability biomarkers and continuous weighted food sensitivity score (*n* = 111).

Exposure: continuous weighted food sensitivity score	LPS IgA β [95% CI] *p*-value	LPS IgG β [95% CI] *p*-value	LPS IgM β [95% CI] *p*-value	Occludin IgA β [95% CI] *p*-value	Occludin IgG β [95% CI] *p*-value	Occludin IgM β [95% CI] *p*-value	CdtB IgG β [95% CI] *p*-value	Vinculin IgG β [95% CI] *p*-value
Model 1′	0.0011 [0.000032,0.0022] (*p* = 0.044)*	0.0013 [0.00061, 0.0019] (*p* < 0.001)*	0.0005 [−0.00077, 0.0018] (*p* = 0.44)	0.0009 [0.000075, 0.0017] (*p* = 0.033)*	0.0013 [0.00045, 0.0022] (*p* = 0.003)*	0.00071 [−0.00046, 0.0019] (*p* = 0.23)	−0.0007 [−0.0029, 0.0015] (*p* = 0.52)	−0.0014 [−0.0033, 0.00062] (*p* = 0.18)
Model 2′	0.00098 [−0.00008, 0.002] (*p* = 0.07)	0.0012 [0.00059, 0.0019] (*p* < 0.001)*	0.0005 [−0.00067, 0.0017] (*p* = 0.40)	0.0008 [0.000015, 0.0017] (*p* = 0.046)*	0.0014 [0.00051, 0.0022] (*p* = 0.002)*	0.00066 [−0.00048, 0.0018] (*p* = 0.25)	−0.00064 [−0.0029, 0.0016] (*p* = 0.57)	−0.0013 [−0.0033, 0.00068] (*p* = 0.19)
Model 3′	0.00082 [−0.0003, 0.0019] (*p* = 0.15)	0.0012 [0.00055, 0.0019] (*p* < 0.001)*	0.00066 [−0.00056, 0.0019] (*p* = 0.29)	0.00071 [−0.00015, 0.0016] (*p* = 0.02)*	0.0012 [0.00029, 0.0021] (*p* = 0.01)*	0.0008 [−0.00039, 0.002] (*p* = 0.19)	−0.00078 [−0.0031, 0.0015] (*p* = 0.51)	−0.0014 [−0.0035, 0.00074] (*p* = 0.20)
Model 4′	−0.018 [−0.053, 0.016] (*p* = 0.29)	0.014 [−0.0064, 0.035] (*p* = 0.17)	0.0021 [−0.036, 0.04] (*p* = 0.91)	0.00092 [−0.00024, 0.0021] (*p* = 0.12)	0.0002 [−0.00098, 0.0014] (*p* = 0.73)	0.00089 [−0.04, 0.033] (*p* = 0.271)	−0.0014 [−0.0045, 0.0017] (*p* = 0.374)	−0.00075 [−0.0036, 0.0021] (*p* = 0.60)

Model 1 = Intestinal permeability biomarker only; Model 2 = Model 1 + age + sex; Model 3 = Model 2 + consumption of wheat, dairy, and eggs; Model 4 = Model 3 + severity of the reaction to wheat, dairy, and eggs. (p-value < 0.05)*.

## Discussion

The results of these analyses indicate a positive and significant association between IgG-mediated food sensitivities and intestinal permeability biomarkers, as indicated by anti-LPS and anti-occludin IgG and IgA antibodies. After the statistical models were adjusted for the severity of the reactions to commonly reactive foods (e.g., wheat, dairy, and eggs), however, the significance of the associations was attenuated. This suggests while other food sensitivities were present, the relationship with increased intestinal permeability biomarkers may be largely due to the severity of IgG-mediated immune reactions against wheat, dairy, and eggs specifically, instead of the presence of food sensitivities generally. Additionally, no differences in average concentrations of permeability biomarkers or antibodies against commonly reactive foods were observed between symptomatic and asymptomatic individuals, suggesting that perhaps asymptomatic individuals with IgG-mediated food sensitivities to commonly reactive foods may have increased intestinal permeability even in the absence of current clinical symptoms.

Previous studies have revealed associations between food-specific IgG titers and certain gastrointestinal pathologies, such as irritable bowel syndrome (IBS) (20, 21) and inflammatory bowel disease (22), highlighting a connection between IgG-mediated food sensitivities and gastrointestinal inflammation. Pro-inflammatory cytokines produced during these events may act directly on the intestinal barrier to increase permeability (23, 24). Independent of underlying inflammatory pathology or isolated events, certain dietary components are known increase permeability under normal conditions. Gliadin, for example, is a component of wheat gluten which increases production of the protein zonulin (25). Zonulin signaling subsequently increases intestinal permeability by triggering enterocyte tight junction disassembly, an activity that occurs irrespective of a food sensitivity response (26, 27). Since the associations between food sensitivities and anti-LPS and anti-occludin antibodies in our study lose significance when the severity of the wheat, dairy, and eggs reactions were adjusted for, the observed relationship may be a result of the cumulative effects of gliadin-induced zonulin (25) and food sensitivity-induced inflammatory factors [e.g., cytokines (23, 24)] acting concurrently on intestinal permeability, as opposed to either factor alone.

Notably, despite molecular similarities between some dairy proteins and gluten, evidence suggests that dairy proteins may not impact permeability in a zonulin-mediated fashion, an instead may be mediated through immune mechanisms. In patients with celiac disease, for example, cow’s milk protein elicited inflammatory responses, such as increased neutrophil activation (28). However, in healthy adult males, no associations were found between a short-term high dairy diet and permeability biomarkers, including serum zonulin (29). Additionally, an *in vitro* model of intestinal permeability demonstrated that while gliadin-exposed Caco-2 cells released zonulin, casein-exposed cells did not (30).

The relationship between egg consumption and intestinal permeability is less clear than that of dairy, yet similarly appears to be independent of zonulin. A recent study looking at the relationship between food-specific IgG titers and serum zonulin levels in patients with irritable bowel syndrome did not find any significant correlations between egg-specific antibodies and zonulin (20). As such, the contributions of egg and dairy consumption to intestinal permeability, specifically in the context of IgG-mediate food sensitivities, remain unclear.

Additionally, states of prolonged inflammation, such as those that may occur during hypersensitivity responses to food antigens, can create an environment where antibodies can be generated against local self-antigens (e.g., occludin) (2). As occludin is a protein involved in the formation of the tight junctions between enterocytes (7, 8), antibodies produced against occludin may lead to further barrier dysfunction through antibody-mediated mechanisms (31) and could subsequently contribute to the enhanced translocation of LPS and CdtB across intestinal barrier. While both systemic LPS and CdtB exposure can trigger immune responses (32–38) and possibly contribute to inflammatory-mediated barrier permeability, LPS specifically can directly increase intestinal permeability through TLR4-MyD88-dependant mechanisms (39).

Although we observed associations between food sensitivities and titers of anti-LPS and anti-occludin antibodies, none were observed with that of anti-CdtB and anti-vinculin antibodies. The presence of anti-CdtB and anti-vinculin have been previously linked to certain gastrointestinal pathologies such as IBS, and may be enhanced following infectious gastroenteritis (10, 15, 40–43). CdtB is the active component of Cdt, a toxin produced by numerous Gram-negative pathogenic bacteria (44). Similarly to LPS, CdtB may cross the intestinal barrier during states of increased intestinal permeability and elicit an immune response, resulting in anti-CdtB antibody production. Due to molecular mimicry, anti-CdtB antibodies have the ability to cross-react with vinculin, a cytoskeletal protein which works to maintain cellular adhesion; autoantibodies may also be produced against vinculin specifically (13, 40). Moreover, CdtB itself may directly impact vinculin function, further disrupting barrier integrity (45).

We originally hypothesized that anti-CdtB and anti-vinculin antibodies may also act as biomarkers for increased intestinal permeability following inflammation induced by IgG-mediated food sensitivities, as this increased permeability may allow bacterial-derived antigens to cross into systemic circulation. As there were no significant associations observed between IgG-mediated food sensitivities and anti-CdtB and anti-vinculin antibody titers, we hypothesize that in the absence of an infection with specific CdtB-producing pathogenic bacteria, concentrations of CdtB are perhaps not elevated to such a degree that would induce antibody production upon translocation.

Regarding the concentrations of food-specific IgG antibodies in symptomatic and asymptomatic individuals, existing evidence also highlights the presence of food-specific IgG in both symptomatic and asymptomatic individuals (46). The lack of difference in IgG antibody titers and intestinal permeability biomarkers between symptomatic and asymptomatic individuals may also be due to participants not currently ingesting the food items to which they have sensitivities, although this cannot be confirmed without the use of a food frequency questionnaire. Previous studies show that elimination of foods based on IgG food sensitivity test results also reduced gastrointestinal symptoms associated with IBS (47–49), indicating that individuals may still have food sensitivities but a lack of clinical symptoms if they are not actively ingesting foods to which they are sensitive.

### Clinical relevance

This research has three important clinical implications. First, our results suggest that the presence of food IgG antibodies, rather than presentation of symptoms, is perhaps more indicative of concurrently increased intestinal permeability, and that in some cases these IgG-mediated reactions reach clinical significance by producing symptoms and in other cases not. We propose that intestinal permeability biomarkers, and thus perhaps increased intestinal permeability, may be present in absence of clinical GI symptoms if a pathology such as food sensitivities is present. Thus, when clinically assessing IgG food sensitivities or intestinal permeability, the status of GI symptoms (i.e., symptomatic vs. asymptomatic) of an individual should not rule out the possibility of increased permeability. Due to increasing evidence highlighting the connection between increased intestinal permeability and many chronic diseases (50), this may have implications for prevention-oriented clinical practice.

Second, this study utilizes two intestinal permeability biomarkers, anti-CdtB, and anti-vinculin antibodies, which have not yet been used in the context of IgG-mediated food sensitivities. As such, this research adds to the growing body of literature surrounding their associations with different gastrointestinal pathologies and subsequently, their context-dependent utility as intestinal permeability biomarkers.

Finally, this data suggests that clinicians ordering IgG food testing should also consider that increased permeability may be present in patients whose IgG food tests come back with positive results, especially regarding individuals with severe reactions to wheat, dairy, or eggs; Conversely, as directionality of the relationship between IgG food sensitivities and intestinal permeability biomarkers cannot be determined with the cross-sectional study design, food sensitivities should also be considered when clinically assessing either potential causes or physiological repercussions of intestinal permeability.

### Strengths and limitations

A notable strength of this study is the evaluation of associations in a sizable sample of humans, in both symptomatic and asymptomatic individuals, allowing to test whether these biomarkers are specifically related to clinical symptom presentation. This research also sheds light on the potential clinical utility of IgG food antibody testing, which is controversial in numerous specialties because of historic inconsistencies with the mechanisms, effects and presence of food-specific IgG in both symptomatic and asymptomatic individuals (46). Moreover, we provide insight into a possible connection between IgG food sensitivities and increased intestinal permeability biomarkers.

A major limitation includes the cross-sectional study design, which makes assessing the directionality of the relationship impossible. Further research is necessary to fully understand whether food sensitives are a contributor to or an effect of increased intestinal permeability.

Additionally, we acknowledge that the absence of BMI from either the inclusion or exclusion criteria, or as a covariate in statistical analyses, may be a limitation. However, based on existing literature we do not believe that this absence confounds the results in the context of this study. Evidence suggests that associations between BMI and intestinal permeability biomarkers may occur context of an unhealthy metabolic profile, which includes other factors such as hyperglycemia and hyperlipidemia (51). In our own study, we specifically exclude participants with hyperglycemia and other physiological conditions which often coincide with metabolic risk factors. Additionally, associations between intestinal permeability biomarkers and BMI may not only be dependent on other existing metabolic risk factors, but also on the specific type of biomarker being measured and the mechanism of permeability (e.g., transcellular versus paracellular) (51–53); moreover, these associations in healthy versus obese populations are not consistent across studies.

## Conclusion

Our results suggest that elevated food-specific IgG antibodies may be present in conjunction with increased concentrations intestinal permeability biomarkers, and those common highly reactive foods like wheat, dairy, and eggs are the foods may drive the relationship between elevated IgG antibodies and increased intestinal permeability biomarkers, irrespective of current clinical symptoms. Additionally, this study suggests that IgG-mediated food sensitivities to commonly reactive foods (e.g., wheat, eggs, and dairy) can co-occur with the production of antibodies against self-antigens, such as occludin. Accordingly, we propose that food sensitives should be considered when clinically assessing intestinal permeability, either as a potential cause of or effect of changes to the integrity of the intestinal barrier function. However, further research is needed to fully determine the etiology of IgG-mediated food sensitivities and the full extent of its pathological implications in order to better develop potential treatment options.

## Data availability statement

The original contributions presented in this study are included in the article/supplementary material, further inquiries can be directed to the corresponding author.

## Ethics statement

The studies involving human participants were reviewed and approved by the Institutional Review Board of the National University of Natural Medicine (IRB # 102114). The patients/participants provided their written informed consent to participate in this study.

## Author contributions

AV and RB were involved in the conceptualization, methodology, and formal analysis. AV was involved in writing the original manuscript draft and visualization of data. All authors involved in manuscript review and editing, read, and agreed to the published version of the manuscript.
